# (*E*)-*N*-[(1,3-Dihydro­naphtho[2,3-*c*]furan-4-yl)phenyl­methyl­ene]aniline

**DOI:** 10.1107/S1600536809041889

**Published:** 2009-10-17

**Authors:** Ning-De He, Wei Wang

**Affiliations:** a Department of Chemistry, Shaoxing University, Shaoxing 312000, People’s Republic of China; b Yancheng Institute of Technology, School of Chemical and Biological Engineering, Yancheng 224003, People’s Republic of China

## Abstract

The title compound, C_25_H_19_NO was synthesized by a Pd-catalysed intra­molecular Diels–Alders reaction. The dihedral angle between the two benzene rings is 82.33 (5)° and the dihedral angles between the hydro­naphtho[2,3-*c*]furan plane and the two benzene rings are 89.50 (3) and 77.64 (2)°. The O atom is displaced by 0.5929 (3) Å from the hydro­naphtho[2,3-*c*]furan plane.

## Related literature

For Pd-catalysed [2 + 2 + 2] cocyclization of diynes and arynes, see: Sato *et al.* (2004 ▶, 2007 ▶). For the biological activity of hydro­naphtho[2,3-*c*]furan derivatives, see: Baldwin *et al.* (1995 ▶); Takadoi *et al.* (1999 ▶). For bond-length data, see: Allen *et al.* (1987 ▶).
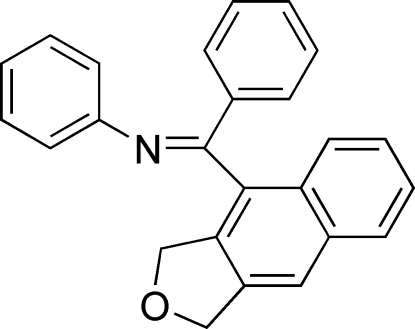


## Experimental

### 

#### Crystal data

C_25_H_19_NO
*M* *_r_* = 349.41
Triclinic, 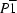

*a* = 9.326 (2) Å
*b* = 10.198 (2) Å
*c* = 10.878 (2) Å
α = 64.410 (10)°
β = 79.037 (11)°
γ = 86.299 (12)°
*V* = 915.9 (3) Å^3^
*Z* = 2
Mo *K*α radiation
μ = 0.08 mm^−1^
*T* = 293 K
0.27 × 0.25 × 0.19 mm

#### Data collection

Bruker SMART CCD area-detector diffractometer
Absorption correction: multi-scan (*SADABS*; Bruker, 2000 ▶) *T*_min_ = 0.979, *T*_max_ = 0.985
4805 measured reflections
3227 independent reflections
1706 reflections with *I* > 2σ(*I*)
*R*_int_ = 0.046

#### Refinement

*R*[*F* ^2^ > 2σ(*F* ^2^)] = 0.105
*wR*(*F* ^2^) = 0.278
*S* = 1.04
3227 reflections
244 parameters
H-atom parameters constrained
Δρ_max_ = 0.59 e Å^−3^
Δρ_min_ = −0.48 e Å^−3^

### 

Data collection: *SMART* (Bruker, 2000 ▶); cell refinement: *SAINT* (Bruker, 2000 ▶); data reduction: *SAINT*; program(s) used to solve structure: *SHELXS97* (Sheldrick, 2008 ▶); program(s) used to refine structure: *SHELXL97* (Sheldrick, 2008 ▶); molecular graphics: *SHELXTL* (Sheldrick, 2008 ▶); software used to prepare material for publication: *SHELXTL*.

## Supplementary Material

Crystal structure: contains datablocks I, global. DOI: 10.1107/S1600536809041889/hg2580sup1.cif

Structure factors: contains datablocks I. DOI: 10.1107/S1600536809041889/hg2580Isup2.hkl

Additional supplementary materials:  crystallographic information; 3D view; checkCIF report

## Figures and Tables

**Fig. 1.**
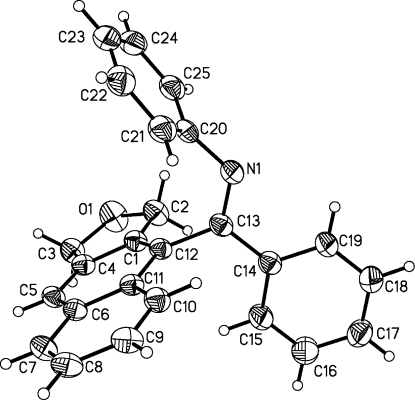
The independent molecules of the title compound, showing 30% probability displacement ellipsoids and the atom-numbering scheme.

## supplementary crystallographic information

### Comment

Hydronaphtho [2,3-*c*]-furan derivatives exhibit potent and selective antagonism against muscarine M~2~ receptor and are expected for the use as a therapeutic drug of Arzheimer's disease for example himbacine (Baldwin *et al.*,1995; Takadoi *et al.*, 1999). These compounds were syhthesized by Pd Catalyzed [2 + 2+2] cocyclization of diynes and arynes(Sato *et al.*, 2004, 2007). We report here the synthesis and crystal structure of the title compound. In the structure of title compound(Fig1), the values of the geometric parameters in (I) are normal (Allen *et al.*, 1987) (Table 1). The intramolecular dihedral angle between the two benzene rings is 82.33 (5)°. The dihedral angles between the hydronaphtho[2,3-*c*]furan plane and the two benzene planes are 89.50 (3)° and 77.64 (2)°. The distance of O1 to the hydronaphtho[2,3-*c*]furan plane is 0.5929 (3) Å

### Experimental

To a solution of Pd(dba)_3_.CHCl_3_ (20.8 mg, 0.02 mmol) in 2.0 ml anhydrous DMF under argon was added 1a (139.6 mg, 0.4 mmol), triethylamine (60.6 mg, 0.6 mmol). The mixture was stirred at 120 for 8 h. The reaction was quenched with a saturated aqueous solution of ammonium chloride, and the mixture was extracted with Et_2_O.The combined organic extracts were washed with water and saturated brine. The organic layer was dried (Na_2_SO_4_) and concentrated *in vacuo*. The residue was purified by chromatography on silica gel. The resulting solution was vapor at room temperature for 6 d, after which block-shaped crystals of the title compound suitable for X-ray diffraction analysis were obtained, yield 80%.

### Refinement

The H atoms were fixed geometrically and were treated as riding on their parent C atoms, with C—H distances in the range of 0.93–0.97 Å (methanol hydroxyl) and with *U*ĩso~(H) = 1.2*U*~eq~(parent atom),.

### Crystal data

**Table:**

C_25_H_19_NO	*Z* = 2
*M**_r_* = 349.41	*F*(000) = 368
Triclinic, *P*1	*D*_x_ = 1.267 Mg m^−^^3^
Hall symbol: -P 1	Mo *K*α radiation, λ = 0.71073 Å
*a* = 9.326 (2) Å	Cell parameters from 831 reflections
*b* = 10.198 (2) Å	θ = 2.7–28.6°
*c* = 10.878 (2) Å	µ = 0.08 mm^−^^1^
α = 64.41 (1)°	*T* = 293 K
β = 79.037 (11)°	Block, yellow
γ = 86.299 (12)°	0.27 × 0.25 × 0.19 mm
*V* = 915.9 (3) Å^3^	

### Data collection

**Table:**

Bruker SMART CCD area-detector diffractometer	3227 independent reflections
Radiation source: fine-focus sealed tube	1706 reflections with *I* > 2σ(*I*)
graphite	*R*_int_ = 0.046
phi and ω scans	θ_max_ = 25.1°, θ_min_ = 2.1°
Absorption correction: multi-scan (*SADABS*; Bruker, 2000)	*h* = −10→11
*T*_min_ = 0.979, *T*_max_ = 0.985	*k* = −12→11
4805 measured reflections	*l* = −12→12

### Refinement

**Table:**

Refinement on *F*^2^	Primary atom site location: structure-invariant direct methods
Least-squares matrix: full	Secondary atom site location: difference Fourier map
*R*[*F*^2^ > 2σ(*F*^2^)] = 0.105	Hydrogen site location: inferred from neighbouring sites
*wR*(*F*^2^) = 0.278	H-atom parameters constrained
*S* = 1.04	*w* = 1/[σ^2^(*F*_o_^2^) + (0.150*P*)^2^] where *P* = (*F*_o_^2^ + 2*F*_c_^2^)/3
3227 reflections	(Δ/σ)_max_ < 0.001
244 parameters	Δρ_max_ = 0.59 e Å^−^^3^
0 restraints	Δρ_min_ = −0.48 e Å^−^^3^

### Special details

**Table:**

Geometry. All e.s.d.'s (except the e.s.d. in the dihedral angle between two l.s. planes) are estimated using the full covariance matrix. The cell e.s.d.'s are taken into account individually in the estimation of e.s.d.'s in distances, angles and torsion angles; correlations between e.s.d.'s in cell parameters are only used when they are defined by crystal symmetry. An approximate (isotropic) treatment of cell e.s.d.'s is used for estimating e.s.d.'s involving l.s. planes.
Refinement. Refinement of *F*^2^ against ALL reflections. The weighted *R*-factor *wR* and goodness of fit *S* are based on *F*^2^, conventional *R*-factors *R* are based on *F*, with *F* set to zero for negative *F*^2^. The threshold expression of *F*^2^ > σ(*F*^2^) is used only for calculating *R*-factors(gt) *etc*. and is not relevant to the choice of reflections for refinement. *R*-factors based on *F*^2^ are statistically about twice as large as those based on *F*, and *R*- factors based on ALL data will be even larger.

### Fractional atomic coordinates and isotropic or equivalent isotropic displacement parameters (Å^2^)

**Table:**

	*x*	*y*	*z*	*U*_iso_*/*U*_eq_	
C11	0.1356 (4)	0.2470 (4)	0.7731 (4)	0.0433 (9)	
C12	0.1319 (4)	0.1578 (4)	0.7038 (4)	0.0435 (9)	
C6	0.1621 (4)	0.1824 (4)	0.9125 (4)	0.0446 (9)	
C5	0.1846 (4)	0.0328 (4)	0.9765 (4)	0.0486 (10)	
H5	0.1995	−0.0101	1.0677	0.058*	
C4	0.1851 (4)	−0.0506 (4)	0.9077 (4)	0.0471 (9)	
N1	−0.0090 (4)	0.2261 (4)	0.5151 (3)	0.0583 (10)	
C1	0.1585 (4)	0.0116 (4)	0.7703 (4)	0.0464 (10)	
C10	0.1162 (4)	0.3989 (4)	0.7104 (4)	0.0529 (10)	
H10	0.0978	0.4427	0.6204	0.064*	
C13	0.1141 (4)	0.2222 (4)	0.5537 (4)	0.0473 (10)	
C7	0.1645 (4)	0.2734 (4)	0.9793 (4)	0.0553 (11)	
H7	0.1788	0.2320	1.0707	0.066*	
C14	0.2449 (4)	0.2815 (4)	0.4463 (4)	0.0495 (10)	
C9	0.1235 (4)	0.4827 (4)	0.7779 (4)	0.0585 (11)	
H9	0.1131	0.5828	0.7328	0.070*	
C20	−0.1432 (4)	0.1792 (5)	0.6102 (4)	0.0527 (11)	
C8	0.1467 (5)	0.4190 (5)	0.9153 (5)	0.0605 (11)	
H8	0.1499	0.4762	0.9621	0.073*	
C19	0.2363 (5)	0.3438 (5)	0.3073 (4)	0.0600 (11)	
H19	0.1462	0.3476	0.2813	0.072*	
C18	0.3576 (5)	0.3999 (5)	0.2072 (5)	0.0708 (13)	
H18	0.3490	0.4425	0.1142	0.085*	
C21	−0.2194 (5)	0.2719 (5)	0.6612 (4)	0.0632 (12)	
H21	−0.1773	0.3600	0.6434	0.076*	
C2	0.1712 (5)	−0.1041 (4)	0.7202 (4)	0.0645 (12)	
H2A	0.0832	−0.1104	0.6875	0.077*	
H2B	0.2538	−0.0840	0.6450	0.077*	
C25	−0.2089 (5)	0.0486 (5)	0.6375 (4)	0.0626 (12)	
H25	−0.1595	−0.0133	0.6021	0.075*	
O1	0.1914 (4)	−0.2359 (3)	0.8365 (3)	0.0819 (11)	
C22	−0.3594 (5)	0.2322 (5)	0.7394 (5)	0.0699 (13)	
H22	−0.4112	0.2944	0.7732	0.084*	
C3	0.2152 (5)	−0.2078 (4)	0.9480 (4)	0.0608 (11)	
H3A	0.3153	−0.2286	0.9625	0.073*	
H3B	0.1500	−0.2676	1.0328	0.073*	
C24	−0.3477 (5)	0.0100 (6)	0.7172 (5)	0.0705 (13)	
H24	−0.3902	−0.0784	0.7367	0.085*	
C23	−0.4213 (5)	0.1018 (6)	0.7669 (5)	0.0718 (14)	
H23	−0.5145	0.0758	0.8199	0.086*	
C15	0.3807 (5)	0.2772 (5)	0.4806 (5)	0.0739 (14)	
H15	0.3896	0.2365	0.5734	0.089*	
C16	0.5024 (5)	0.3318 (6)	0.3804 (5)	0.0892 (17)	
H16	0.5928	0.3263	0.4064	0.107*	
C17	0.4933 (5)	0.3938 (5)	0.2436 (5)	0.0753 (14)	
H17	0.5763	0.4313	0.1760	0.090*	

### Atomic displacement parameters (Å^2^)

**Table:**

	*U*^11^	*U*^22^	*U*^33^	*U*^12^	*U*^13^	*U*^23^
C11	0.041 (2)	0.038 (2)	0.050 (2)	0.0026 (15)	−0.0135 (17)	−0.0164 (17)
C12	0.041 (2)	0.043 (2)	0.046 (2)	0.0045 (16)	−0.0145 (16)	−0.0168 (17)
C6	0.040 (2)	0.046 (2)	0.050 (2)	0.0062 (16)	−0.0150 (17)	−0.0201 (17)
C5	0.048 (2)	0.050 (2)	0.048 (2)	0.0031 (17)	−0.0200 (17)	−0.0163 (18)
C4	0.044 (2)	0.041 (2)	0.054 (2)	0.0055 (16)	−0.0168 (17)	−0.0169 (17)
N1	0.052 (2)	0.069 (2)	0.0467 (19)	0.0011 (17)	−0.0183 (16)	−0.0149 (16)
C1	0.046 (2)	0.042 (2)	0.051 (2)	0.0049 (17)	−0.0155 (18)	−0.0181 (17)
C10	0.054 (3)	0.048 (2)	0.054 (2)	0.0031 (18)	−0.0186 (19)	−0.0156 (19)
C13	0.054 (2)	0.040 (2)	0.049 (2)	0.0058 (17)	−0.0199 (19)	−0.0169 (17)
C7	0.058 (3)	0.056 (3)	0.059 (2)	0.008 (2)	−0.024 (2)	−0.027 (2)
C14	0.055 (3)	0.044 (2)	0.050 (2)	0.0060 (18)	−0.0173 (19)	−0.0176 (17)
C9	0.057 (3)	0.044 (2)	0.077 (3)	0.0072 (19)	−0.026 (2)	−0.024 (2)
C20	0.052 (3)	0.058 (3)	0.043 (2)	0.007 (2)	−0.0249 (19)	−0.0111 (18)
C8	0.062 (3)	0.053 (3)	0.077 (3)	0.001 (2)	−0.022 (2)	−0.034 (2)
C19	0.058 (3)	0.068 (3)	0.048 (2)	0.002 (2)	−0.018 (2)	−0.016 (2)
C18	0.066 (3)	0.080 (3)	0.054 (3)	−0.005 (2)	−0.012 (2)	−0.016 (2)
C21	0.062 (3)	0.062 (3)	0.063 (3)	0.007 (2)	−0.023 (2)	−0.019 (2)
C2	0.079 (3)	0.052 (3)	0.067 (3)	0.012 (2)	−0.028 (2)	−0.025 (2)
C25	0.065 (3)	0.063 (3)	0.057 (3)	0.007 (2)	−0.028 (2)	−0.017 (2)
O1	0.120 (3)	0.0447 (17)	0.085 (2)	0.0196 (17)	−0.0338 (19)	−0.0285 (16)
C22	0.065 (3)	0.076 (3)	0.064 (3)	0.019 (3)	−0.021 (2)	−0.024 (2)
C3	0.071 (3)	0.049 (2)	0.061 (3)	0.010 (2)	−0.023 (2)	−0.019 (2)
C24	0.065 (3)	0.076 (3)	0.067 (3)	−0.005 (3)	−0.025 (2)	−0.021 (2)
C23	0.055 (3)	0.089 (4)	0.062 (3)	0.001 (3)	−0.023 (2)	−0.019 (3)
C15	0.057 (3)	0.098 (4)	0.055 (3)	0.000 (2)	−0.021 (2)	−0.017 (2)
C16	0.049 (3)	0.129 (5)	0.071 (3)	−0.002 (3)	−0.015 (2)	−0.024 (3)
C17	0.061 (3)	0.084 (3)	0.066 (3)	−0.006 (2)	−0.002 (2)	−0.021 (3)

### Geometric parameters (Å, °)

**Table:**

C11—C10	1.413 (5)	C8—H8	0.9300
C11—C12	1.416 (5)	C19—C18	1.367 (6)
C11—C6	1.432 (5)	C19—H19	0.9300
C12—C1	1.377 (5)	C18—C17	1.387 (6)
C12—C13	1.513 (5)	C18—H18	0.9300
C6—C5	1.398 (5)	C21—C22	1.393 (6)
C6—C7	1.407 (5)	C21—H21	0.9300
C5—C4	1.353 (5)	C2—O1	1.426 (5)
C5—H5	0.9300	C2—H2A	0.9700
C4—C1	1.414 (5)	C2—H2B	0.9700
C4—C3	1.491 (5)	C25—C24	1.388 (6)
N1—C13	1.288 (5)	C25—H25	0.9300
N1—C20	1.422 (5)	O1—C3	1.419 (5)
C1—C2	1.489 (5)	C22—C23	1.372 (7)
C10—C9	1.357 (5)	C22—H22	0.9300
C10—H10	0.9300	C3—H3A	0.9700
C13—C14	1.470 (5)	C3—H3B	0.9700
C7—C8	1.355 (6)	C24—C23	1.361 (6)
C7—H7	0.9300	C24—H24	0.9300
C14—C15	1.379 (5)	C23—H23	0.9300
C14—C19	1.381 (5)	C15—C16	1.368 (6)
C9—C8	1.404 (6)	C15—H15	0.9300
C9—H9	0.9300	C16—C17	1.360 (6)
C20—C21	1.387 (6)	C16—H16	0.9300
C20—C25	1.390 (6)	C17—H17	0.9300
			
C10—C11—C12	123.0 (3)	C14—C19—H19	119.3
C10—C11—C6	117.8 (3)	C19—C18—C17	120.3 (4)
C12—C11—C6	119.3 (3)	C19—C18—H18	119.8
C1—C12—C11	119.3 (3)	C17—C18—H18	119.8
C1—C12—C13	119.3 (3)	C20—C21—C22	119.5 (4)
C11—C12—C13	121.2 (3)	C20—C21—H21	120.2
C5—C6—C7	122.7 (4)	C22—C21—H21	120.2
C5—C6—C11	119.2 (3)	O1—C2—C1	105.7 (3)
C7—C6—C11	118.2 (3)	O1—C2—H2A	110.6
C4—C5—C6	120.9 (4)	C1—C2—H2A	110.6
C4—C5—H5	119.5	O1—C2—H2B	110.6
C6—C5—H5	119.5	C1—C2—H2B	110.6
C5—C4—C1	120.5 (3)	H2A—C2—H2B	108.7
C5—C4—C3	131.5 (4)	C24—C25—C20	120.5 (4)
C1—C4—C3	108.0 (3)	C24—C25—H25	119.8
C13—N1—C20	122.9 (3)	C20—C25—H25	119.8
C12—C1—C4	120.8 (3)	C3—O1—C2	111.0 (3)
C12—C1—C2	130.7 (3)	C23—C22—C21	120.4 (5)
C4—C1—C2	108.4 (3)	C23—C22—H22	119.8
C9—C10—C11	121.9 (4)	C21—C22—H22	119.8
C9—C10—H10	119.1	O1—C3—C4	106.0 (3)
C11—C10—H10	119.1	O1—C3—H3A	110.5
N1—C13—C14	118.4 (3)	C4—C3—H3A	110.5
N1—C13—C12	123.5 (3)	O1—C3—H3B	110.5
C14—C13—C12	118.1 (3)	C4—C3—H3B	110.5
C8—C7—C6	122.4 (4)	H3A—C3—H3B	108.7
C8—C7—H7	118.8	C23—C24—C25	119.9 (5)
C6—C7—H7	118.8	C23—C24—H24	120.1
C15—C14—C19	117.5 (4)	C25—C24—H24	120.1
C15—C14—C13	121.3 (4)	C24—C23—C22	120.6 (5)
C19—C14—C13	121.2 (4)	C24—C23—H23	119.7
C10—C9—C8	120.3 (4)	C22—C23—H23	119.7
C10—C9—H9	119.8	C16—C15—C14	121.2 (4)
C8—C9—H9	119.8	C16—C15—H15	119.4
C21—C20—C25	119.1 (4)	C14—C15—H15	119.4
C21—C20—N1	120.2 (4)	C17—C16—C15	121.1 (5)
C25—C20—N1	120.0 (4)	C17—C16—H16	119.4
C7—C8—C9	119.4 (4)	C15—C16—H16	119.4
C7—C8—H8	120.3	C16—C17—C18	118.5 (4)
C9—C8—H8	120.3	C16—C17—H17	120.7
C18—C19—C14	121.3 (4)	C18—C17—H17	120.7
C18—C19—H19	119.3		
			
C10—C11—C12—C1	177.0 (3)	C12—C13—C14—C15	0.8 (5)
C6—C11—C12—C1	−2.0 (5)	N1—C13—C14—C19	1.9 (6)
C10—C11—C12—C13	3.1 (5)	C12—C13—C14—C19	−179.1 (3)
C6—C11—C12—C13	−176.0 (3)	C11—C10—C9—C8	−1.9 (6)
C10—C11—C6—C5	−178.7 (3)	C13—N1—C20—C21	82.6 (5)
C12—C11—C6—C5	0.4 (5)	C13—N1—C20—C25	−106.4 (5)
C10—C11—C6—C7	1.2 (5)	C6—C7—C8—C9	0.6 (6)
C12—C11—C6—C7	−179.7 (3)	C10—C9—C8—C7	1.2 (6)
C7—C6—C5—C4	−178.3 (3)	C15—C14—C19—C18	−0.4 (6)
C11—C6—C5—C4	1.5 (5)	C13—C14—C19—C18	179.5 (4)
C6—C5—C4—C1	−1.8 (6)	C14—C19—C18—C17	0.8 (7)
C6—C5—C4—C3	175.7 (4)	C25—C20—C21—C22	0.4 (6)
C11—C12—C1—C4	1.8 (5)	N1—C20—C21—C22	171.5 (4)
C13—C12—C1—C4	175.9 (3)	C12—C1—C2—O1	−176.9 (4)
C11—C12—C1—C2	−175.5 (4)	C4—C1—C2—O1	5.5 (4)
C13—C12—C1—C2	−1.4 (6)	C21—C20—C25—C24	−1.3 (6)
C5—C4—C1—C12	0.1 (6)	N1—C20—C25—C24	−172.4 (4)
C3—C4—C1—C12	−177.9 (3)	C1—C2—O1—C3	−9.2 (5)
C5—C4—C1—C2	178.0 (3)	C20—C21—C22—C23	0.5 (6)
C3—C4—C1—C2	−0.1 (4)	C2—O1—C3—C4	9.2 (5)
C12—C11—C10—C9	−178.4 (3)	C5—C4—C3—O1	176.8 (4)
C6—C11—C10—C9	0.7 (6)	C1—C4—C3—O1	−5.5 (4)
C20—N1—C13—C14	−176.4 (3)	C20—C25—C24—C23	1.3 (6)
C20—N1—C13—C12	4.6 (6)	C25—C24—C23—C22	−0.3 (7)
C1—C12—C13—N1	87.9 (5)	C21—C22—C23—C24	−0.6 (7)
C11—C12—C13—N1	−98.2 (4)	C19—C14—C15—C16	−0.4 (7)
C1—C12—C13—C14	−91.2 (4)	C13—C14—C15—C16	179.7 (4)
C11—C12—C13—C14	82.8 (4)	C14—C15—C16—C17	0.9 (8)
C5—C6—C7—C8	178.0 (4)	C15—C16—C17—C18	−0.4 (8)
C11—C6—C7—C8	−1.8 (6)	C19—C18—C17—C16	−0.4 (7)
N1—C13—C14—C15	−178.3 (4)		

## References

[bb1] Allen, F. H., Kennard, O., Watson, D. G., Brammer, L., Orpen, A. G. & Taylor, R. (1987). *J. Chem. Soc., Perkin Trans. 2*, pp. S1–19.

[bb2] Baldwin, J. E., Chesworth, R. A., Parker, J. S. & Russell, A. T. (1995). *Tetrahedron Lett.* **36**, 9551–9554.

[bb3] Bruker (2000). *SADABS*, *SMART* and *SAINT* Bruker AXS Inc., Madison, Wisconsin, USA.

[bb4] Sato, Y., Tamura, T., Kinbara, A. & Mori, M. (2007). *Adv. Synth. Catal.* **349**, 647–661.

[bb5] Sato, Y., Tamura, T. & Mori, M. (2004). *Angew. Chem. Int. Ed* **43**, 2436–2440. 10.1002/anie.200453809 15114584

[bb6] Sheldrick, G. M. (2008). *Acta Cryst.* A**64**, 112–122. 10.1107/S0108767307043930 18156677

[bb7] Takadoi, M., Katoh, T., Ishiwata, A. & Terashima, S. (1999). *Tetrahedron Lett.* **40**, 3399–3402.

